# Dental Hints Department

**Published:** 1907-12

**Authors:** B. H. Teague

**Affiliations:** Aiken, S. C.


### ...DENTAL HINTS DEPARTMENT...

CONDUCTED BY B. H. TEAGUE, D. D. S., AIKEN, S. C., TO WHOM ALL CONTRIBUTIONS TO THIS DEPARTMENT SHOULD BE SENT

The success of a porcelain filling is dependent more upon a correct occlusion and the care exercised in fusing, than upon any other factors in its construction.—Dr. F. T. Van Woert, Cosmico.

Formula for oxpara or formapara cement.—Tricresol, 3; formalin, 1; glycerin, 1—liquid. Zinc oxid, 2; thymol pulv., 1—powder.—Dr. W. H. Jones, Items.

METHOD OF SOLDERING GOLD INLAYS.—In making inlays, whether solid or hollow, pack the matrix, after investing with crystal gold, restoring contours to suit the case.—Dr. F. C. Runge, Summary.

HANDLING FORCEPS.—A few drops of compound tincture of benzoin rubbed on the hand will prevent the forceps handles from slipping, which they are liable to do on sultry and muggy days.—Geo. Ziederbraum, Dental Register.

OBTUNDING SENSITIVE DENTIN.—I am using refrigeration with ethyl-chlorid for the purpose of obtunding sensitive dentin. In applying it I always put some in the cavity on a piece of cotton, which takes the shock away so that there is not so much pain in the after treatment.—Dr. Lemley, Pacific Dental Gazette.

To keep moisture from working through the hole in the rubber turn the edge hugging the tooth toward the gum by the aid of a broad-bladed burnisher, gently working around the neck, turning the edge as desired. If it will not "stay put" dip the burnisher in sandarac varnish.—R. B. Tuller, American Dental Journal.

STERILIZING THE FORCEPS.—The forceps, including the handles, are boiled in a solution of common washing soda. about a quart of water to a piece of the soda the size of a walnut; there is no rust. A mixture of vaelin and carbolic acid rubbed in the joints while hot will keep them as good as new.—F. E. Garner, British Dental Journal.

If you are buying alloys in large quantities you should reduce the amount of mercury as the alloy gets older. A given alloy which requires 7 parts of mercury when it is placed on the market will not require over 6 parts of mercury if you keep it in your office a year, because the alloy has been a little more annealed.—Dr. M. L. Ward, Dental Review.

ARRANGING A LOWER SET OF TEFTH.—In arranging a lower set, commence with the second bicuspids, as four-fifths of the lower anterior teeth are too wide for the uppers, and it is essential that the bicuspids properly occlude. Then use teeth just wide enough to fill the space between the bicuspids.—L. P. Haskell, Dentists' Magazine.

TO REDUCE PAIN IN THE REMOVAL OF CALCULI.—For the purpose of reducing the pain incident to scaling I recommend packing the pocket with a rope of cotton saturated in a one per cent. cocain-adrenalin solution, allowing it to remain five minutes.—Elgin McWhinney, American Dental Journal.

HYPHERTROPHIED TISSUE.—The following mouth wash has proved very valuable in assisting the aborption of hypertrophied tissue:

℞ Zinci Chloridii.................... ....gr v
Auqae Menth prep.................8 3j

—H. Chapman, The Dental Record.

TO TRIM AND STERILIZE COTTON.—In winding cotton on a broach it is apt to be extended a little beyond the end, and if not removed prevents the dressing proper from reaching remote parts of the root. To dispose of this tag end touch it to the flame; sterilization comes with the fire.—Office and Laboratory.

Nearly all of the dentists went to the army to plug Yankees, and perhaps to be filled with lead by the Yanks. We give a Confederate cure for toothache: Take equal quantities of alum and common salt, pulverize and mix and apply to the hollow tooth on a piece of wet cotton. The remedy is very simple, cheap and within the reach of all. If any will try it it will be found infallible.—Charleston, S. C. News and Courier.

Oral Prohpylaxis.—Phenol-Sodique has a great field of usefulness in combination with pulverized pumice stone in the work of oral prophylaxis. Its alkaline reaction together with its stimulative and antiseptic qualities produce the good results that are witnessed almost daily by those engaged in this line of work.—E. T. Loeffler, Dental Register.

Florida State Dental Society.—Officers elected were: Dr. A. S. York, Live Oak, president; Dr. A. B. Phillips, St. Augustine, first vice-president; Dr. George D. Young, St. Augustine, second vice-president; Dr. D. G. Barnett, Arcadia, treasurer; Dr. Carroll H. Frink, Fernandina, corresponding secretary; Dr. R. P. Taylor, Jacksonville, recording secretary.

Taking Impressions.—In cases which are extremely sensitive and easily nauseated, I have found it useful and helpful to sponge the mouth with hydrogen dioxid to clean the mucus surfaces, and then apply a solution of eucain to the whole palate. This will succeed in the most exaggerated cases of palatal sensitivity.—J. B. Hartzell, Texas Dental Journal.

To Clean Wax.—After melting and straining boil the wax for 20 minutes in a solution composed of half-ounce oxalic acid to each quart of water. Old wax may be made cleaner than new. Two drops of oil of cassia added to each pound keeps wax asceptic and render it less unpleasant to the patient.—Thos. Fletcher, Pacific Dental Gazette.

Compound; Compound Impression.—Take impression in the usual way. Remove and cool, then trim about one line over whole surface, lay very soft layer of compound over impression and press quickly into place in the mouth; this gives a very sharp and accurate impression.—Dr. J. B. Jordon, Summary.

Sensitive Cavities.—If many sensitive cavities appear in the same mouth their sensitivity may be much relieved by removing the loose decay and washing them out with a stream of tepid water and inserting a filling of equal parts of zinc oxid and thymol crystals, using as a liquid, zinc sulphate solution. This is one of the most satisfactory materials for holding dressing in cavities that we possess.—Dr. A. E. Webster, Den. Mag.

Obtunding.—If three or four drops of a 1 to 1,000 adrenalin chlorid solution be added to 20 to 30 drops of a two per cent solution of novo cain, the least toxic of these anaesthetics, and injected into the tissues about the apex of a tooth or deeply into the interproximal space on each side, there will be such a profound effect on the pulp and dentin of the tooth that the dentin may be cut or the pulp removed without pain.—Dr. A. E. Webster, Den. Mag.

Pain After Tooth Extractnio.—The extraction of an abscessed tooth is generally followed by great pain. I have found lysol to be the ideal remedy in such conditions, placing it undiluted in the socket. It will relieve the pain immediately, help it to check the hemorrhage, and establish antiseptic conditions in the socket.—G. B. Winter, Dental Era.

Repairing A Hole in Rubber Dam.—Dry both sides and mix a little cement, sufficient to fill the hole, and leave an excess on both sides. Force it through and press the sides together. A small piece of vulcanite, moistened in chloroform, may be manipulated in the same manner with good results.—W. A. Robertson, Review.

EXTRACTION DURING PREGNANCY.—The irritation produced by ulceratiou at the root of a tooth is usually more liable to interrupt pregnancy than the administration of nitrous oxide and the removal of the tooth or establishing free drainage. The author has had gas given for this purpose and has never seen any bad results.—Dr. M. E. Jordon, Era.

MOUTH WASH—

℞ Hydronaphthol..............
Menthol....................aa gr.xxx
Olei gaultheriae...........
Olei cassiae.................aa m iv
Spts. vini rect............. ℥ x
Tinct. capsici.............. ℥ j
Aquae destillatae ad q. s· 3 xx M

—Dr. H. C. Ferris, Cosmos.

SOLIDIFIED FORMALDEHYDE.—The various preparations containing formaldehyde are of great value in the treatment of putrescent pulps and abscessed teeth, either with blind or open sacs, and after an experience of about six years, I take off my hat to solidified formaldehyde, properly used, and right here let me state that too much sealed in the tooth will create more trouble than any drug with which I am familiar. The secret of success is to use too little, rather than too much.—Dr. W. H. Jones, Items.

PERICEMENTITIS.—If, instead of using equal parts of aconite, iodine, and chloroform, you use this prescription:

℞ Tinc. Aconiti (rad)......... ....fluid ounces i

Chloroformi...........................fluid ounces iv

Menthol.......................................... gr. xx you will get excellent results.—J. P. Buckley, Dental Digest.

PREPARING SENSITIVE CAVITIES.—A comparatively painless method of cutting away a large body of sensitive dentin is to have the stones or burs run in water. I am able to do so-called heroic cutting with the stones run in water, so that the water is almost a running stream upon the bur or stone, and it can be run at a high rate of speed.—E. J. Perry, Dental Review.

Burnishing A Gold Plate To A Tooth.—When burnishing a piece of gold to a lingual or other aspect of a tooth, a good practice is to place a piece of silk ribbon over the gold and carry the ribbon around the tooth so as to have both ends meet on the opposite side, and hold them firmly with the fingers, thereby holding the gold plate in place. Under this silk the gold may be burnished with very little trouble. —E. M. S. Fernandez, Dental Review.

Injection of Staphylococus Vaccine.—The writer's actual experience is limited to the following case: "In a patient, a girl of twenty, with pyorrhea alveolaris (Riggs' disease). From the pus welling up from two of the teeth I grew the staphylococcus albus. Her opsonic index to this I found normal. Two inocculations at intervals of two weeks, with two hundred and fifty millions staphylococcus vaccine, produced a complete cure.—Dr. G. B. Webb, Den. Mag.

Tooth Bleaching.—If a brown stain persists apply a dilute solution of oxalic acid and quickly wash out cavity with sodium carbonate and plenty of hot water. Dessicate the dentin with hot air syringe, then saturate the interior dentin with white shellac varnish, harden with hot air and fill with white osteo or line it for insertion of porcelain, gold or amalgam.—G. Fisher, British Dental Journal.

Contouring Seam Crowns.—Fit the festoon band; surround band with plaster and with an engine disk contour plaster as you want the crown. Place on model cup filled with moldine, and fill interior of crown with dry plaster or investment material. Cover model cup with molding ring and pour in Fusalloy. Split Fusalloy mold; remove plaster, replace band and swage to contour.—Dr. D. D. Campbell, Kansas Proceedings.

Troublesome Third Molars.—The most frequent cause of the serious complications attending the eruption of third molars is lack of vertical space. The irritation is usually produced by the contact of the previously erupted molar in the opposite jaw coming in contact with the distended membrane covering the erupting tooth. Careful grinding of the cusps of the previously erupted tooth will usually suffice to reduce the inflammation and reduce the pain, restoring the parts to a healty condition.—W. Kelsey, Dental Record.

A Word of Warning in Desensitizing Dentin.—To those who are in the habit of obtunding sensitive dentin by pressure anesthesia I wish to give a word of warning. This is a dangerous procedure, not only because cocain is a protoplasic poison, but also beause, as the cavity has not been excavated, the agent is forced through infected tissue, and made to carry the products of that infection into the pulp—a condition which cannot, by any line of reasoning that I can figure out, be anything but deleterious to the pulp tissue. It is undoubtedly true that many pulps die, even years afterward, as the result of the injudicious use of pressure anesthesia.—E. McWhinney, The Bur.

A Method of Dividing Plaster Impressions Before Removal from the Mouth.—The method is especially suited for dividing plaster impressions of the mandible in those cases where the posterior tooth has considerably tilted forward, locking the impression. The method consists of tying a wire ligature around each tooth, leaving a long end to each ligature, bringing these over the tops of the teeth and tying them together across the space. A piece of tin foil is then hung from this ligature, and the plaster impression taken with this in situ. This causes the plaster impression to break easily into two portions when it comes to be removed, being nearly divided by the septum of tin foil.—C. E. Combe, British Dental Journal.

An Effective Ligature.—In cases where the clamp is objectionable for the retention of the rubber dam, and where the ordinary floss is not sufficiently bulky to prevent the rubber from drawing over it, a most admirable method of using the ligature is to first pass the floss through two pieces of rubber tubing, one piece for the buccal and one for the lingual side of the tooth. This is much to be preferred to stringing beads on the ligature, or using other means of holding the rubber dam. The tubing should be the smallest size sold at the rubber stores, the kind used for slipping over the bows of spectacles where they rest on the ears. To insure against leakage, drop a little sandarac varnish between the tubing and the enamel on the buccal and lingual sides.—E. M. S. Fernandez, Dental Review.

Ointment for Neuralgia.—

℞ Mentholis..................... ........gr. xij
Cocainae..........................gr. iv
Choloralis................... .....gr. ij
Petrolati.... ...................gr. lxxv—M
Fiat unguentum

Sig.—Apply to the painful part and cover with a gauze bandage, if the neuralgia is perorbital or hemicranial.—Dental Register.

Danger of Zinc in Vulcanizer.—If any member of the profession is following the suggestion of putting zinc in the vulcanizer for the purpose of keeping the flask clean, he will find himself in trouble in a short time.

Zinc in a vulcanizer will, in a short time, decompose hot water, evolving hydrogen; and when there is enough of it to replace the steam, the regulator will be operated by hydrogen pressure and the heat will go down, causing the plate to come out soft at the end of the vulcanizing time.—R. E. Luther, Dental Brief.

Mummifying.—I very seldom attempt to drill out and fill out the roots of the bicuspids and molars, but devitalize, open up the pulp chamber, then open the mouth of the root canals with Dr. J. Leon Williams triangular treamers, wipe out tbe same with perhydrol ten per cent., then with a solution, absolute alcohol 1 oz., mercury bichloride 1 gr., and fill the mouths of the canals and pulp chamber with mummifying paste. The Soderbury formulae with aristol or iodoform is used and the tooth sealed with the filling the case may require.—Dr. W. H. Jones, Items.

Carbolic Acid Antidote.—In a communication to the Lancet Mr. John Maberly draws attention to the efficacy of iodin as an antidote in carbolic poisoning. The first hint as to the antidotal value of iodin was conveyed by the practice of a Middlesex Hospital surgeon of rinsing his hands—numbed with carbolic solution—with iodin water. The effect was almost immediate, the iodin removing the numbed feeling as well as the bleached, crinkled condition of the skin. Since then Mr. Maberly has tried its effect internally in one case where carbolic acid was taken, and in two other cases where Jeyes' fluid (a disinfectant) had been accidentally swallowed. The effect in all cases has been excellent and prompt.—Cosmos.

In Selection of Colors—In the exposed surfaces this is a very important matter, yet one that I think is easily managed. First it must be remembered that the cement when placed under a porcelain filling brings to light all the color there is in it, and makes it appear darker than the one selected. It is necessary to make provision for this, and I know of no better way than to select a color as near that of the tooth as possible, and then add enough plain white to make it about two shades lighter than that required. I know of no fixed rule by which this can be done, so it is largely a matter of experiment and experience with each operator, very much the same as in the combination of several colors—Dr. F. T. Van Woent, Cosmos.

Annealing.—All hard alloys should contain not less than sixty-five per cent of silver. Hard alloys are made rapid, medium or slow-setting, entirely by annealing in hotor boiling water, without any change whatever in the formula; but the same result may be attained by aging in a warm atmosphere, which, of course, takes much longer. The well-annealed or well-aged and thus slow-setting hard alloys, are the best to use, since they require less mercury and the expansion is much less. All hard alloys expand to some extent, but should not exceed an expansion of one point or .001 of an inch.—C. S. Fuller, Items.

Sensitive Root Canals.—Not infrequently root canals are sensitive, even though the pulp be all dead and removed. This sensation comes from prolongation of nerves from the pericemental membrane to the pulp cavity. Many such sensitive root canals are sometimes found in the same patient. I have often mistaken the condition for remaining vitality of the pulp and applied more arsenic, only to find the sensitiveness remaining, or complete destruction of the pericemental membrane. I have tried sulphuric acid, nitric acid, zinc chlorid with doubtful results, but have found success with one application of phenol after failure with other agents.—Dr. A. E. Webster, Den. Mag.

Iodin an Anaesthetic.—The antiseptic value of iodin has been recognized for centuries, but the value of any antiseptic is in proportion to the strength in which it can be used. This agent, in its powdered form, as iodoform and aristol, is a standard in our hospitals. It has a quality which mercury bichlorid does not possess—that of producing the destruction of the capsule of a spore. It has been recently found that the solution of this drug becomes more potent when potassium iodid is combined with it, the latter agent increasing its solubility. The U. S. P. published in 1906 directs the addition of potassium iodid to all tinctures of this drug.—Dr. H. C. Ferris, Cosmos.

Pressure Anesthesia.—In using pressure anesthesia for the painless extirpation of pulps when indicated, I use a method which may not be new, yet I have never seen it published. When the tooth is prepared and the cavity rendered aseptic, I take a P. & D. pellet of cocain 1½ and adrenalin 1-600, roll a few shreds of cotton around same, moisten with local solution, place over the exposure and then instead of using rubber, fill the tooth with temporary stopping. Then apply pressure with a warm instrument toward the point of exposure. I have frequently succeeded with the stopping after I had failed with the rubber.—Dr. W. H. Jones, Items.

Controlling Hypersensitive Palate When Taking Impressions.—In a case where no one had been able to get impressions, the throat and palate being so sensitive, impressions were taken with no unpleasant symptoms whatever after the following treatment: Three powders of chloretone, each containing five grains, were given to patient with instructions to take one upon getting up in the morning; another two hours later, eating a very light breakfast; the third after breakfast, before reporting at the office. Two grains were then given at the time of taking the impressions.—A. E. Franklin, Dental Register.

Ethyl Chlorid Spray.—Ethyl chlorid spray is useful in hyper-sensitive dentin and the devitalization of pulps. The most effective way to use cold on a sensitive cavity is first to begin with a continuous blast of cold air from a compressed air tank, and as sensation to this subsides, the spray may be directed upon the isolated tooth. If the cavity be deep or of great area, it should be filled with dry cotton before the spray is applied. Most sensitive labial and buccal cavities may be controlled by this means. There is always some danger of setting up such a congestion in the pulp that it may not recover. Ethyl chlorid spray on an anterior tooth where the pulp is partly dead and painful to touch will work wonders, or if the pulp is to be removed from a sound tooth ethyl chlorid will desensitize the dentin and the cocain pressure method may be used on the pulp when it is reached.—Dr. A. E. Webster, Den. Mag.

In One Operation if Possible.—When scaling a tooth we should not do this partly today and tell the patient to come again tomorrow to have it completed; do not keep opening the wound by continued scaling or probing. When a surgeon makes a wound he wants it to heal as soon as possible. If he is compelled to open it up again he considers it a bad piece of work. If you scale but one tooth at each sitting do it thoroughly and finally; then you will have done your work on a scientific basis. Don't keep pricking at the gums all the time and thus opening wounds which run the risk of becoming reinfected. This I believe, is one of the great advantages of Dr. Younger's and Dr. Good's methods—they make but one operation. I believe if we shall adopt these surgical methods in connection with the prophylaxis treatment we shall all come to the conclusion that pyorrhea alveolaris is a curable disease. I can almost assure you that pyorrhea is curable if treated along these lines, and that everyone can be successful with it.—Dr. N. S. Hoff, Register.

Pyorrhea from Milk Diet.—Mr. Gaodby gives a very detailed bacteriological analysis of thirty-six cases in which general symptoms were present and describes the cultural characters of the organisms isolated. In certain cases he isolated a lactose fermenting baccillus, and this organism would seem to have suggested to him that the infection in pyorrhea alveolaris may come from contaminated milk. Mr. Goadby also refers to the point that pyorrhea alveolaris is a frequent sequela of infectious disease, during which the patient has for some time been upon a milk diet, and that the disease frequently occurs in several members of the same family, and would seem to consider these points evidence that milk may be the original source of infection.—Dr. G. B. Webb, Den. Mag.

Repairing Bridges.—The following is a method that I have used in repairing bridges where pin facings have been used, by substituting Steele's interchangeable facings for the pin facings.

Grind the pins flush with the body of the bridge and fit to the latter a backing of one of Steele's interchangeable facings which will correspond to the space. Cement the backing to the bridge; then, with the drill of the S. S. W. anchor drill set, drill through the backing into the body of the bridge as far as the drill will allow. One hole at each corner will be all that is necessary. Tap the holes with the anchor tap and screw, and insert a gold anchor screw wire into each hole. Grind the wires flush with the backing, and set the facing in the usual way. A repair made in this manner will be neat and strong.—P. Neff Myers, Cosmos.

Volasem.—The preparation is said to be composed of fluid extract violet, fluid extract strophanthus, fluid extract calabar bean or physostigmine (physostigmine is the active principle of calabar bean). The violet is evidently used to mask the extremely bitter taste and somewhat unpleasant odor of the strophanthin and physostigmine. Strophanthin is one of the most powerful of the heart stimulants and has much the same action as adrenalin, i. e., stimulates the heart and slows the pulse.

In volasem we have a preparation which given in a little water before the operation, assuring the patient that it will prevent an unpleasant effect, has a powerful mental as well as physiological action.—Dr. W. H. Jones, Items.

Guthymol.—Thymol, added to gutta-percha makes a very useful preparation for dental purposes. It possesses very desirable working properties, setting slowly and becoming hard. I have used it successfully as a temporary stopping and filling material and to crowd away overhanging gum margins and to obtain impressions of cavities.

To obtain a satisfactory grade of guthymol add to base-plate gutta-percha a five per cent. solution of thymol and soften the gutta-percha under heat. A mixture of guthymol, oil of cajuput, and a few fibers of asbestos makes an excellent root-canal filling.

When ready to fill a cavity add to the required amount of guthymol a few crystals of thymol and spatulate the mass thoroughly, when it will acquire a degree of pliability which renders its insertion in a tooth-cavity an operation of the simpliest character.—Carlos Zacharias, Cosmos.

Noble Metals Recommended for Appliances.—The President, Dr. H. A. Pullen, made an earnest plea for a more extensive use of the pure metals in making regulating appliances. He pointed out that in gold, with its alloys, and irridio-platinum, the orthodontist may accomplish all his purposes, and he raised a number of questions which he hoped would be more scientifically studied, to the end that true answers may be found within a short time. The almost universal use of German silver is prevalent, in his opinion, partly from habit, and partly because it is more convenient to purchase fixtures ready made, and because until very recently no dealer seemed willing to make appliances other than of German silver. These ready-made bands and arches of course look well when first bought, being handsomely gold plated. But in the mouth this plating frequently disappears very rapidly, and considerable discoloration, if not actual foulness often ensues. Teeth in contact with German silver often show metallic stains which it is quite difficult to remove. Dr. Pullen referred to the oft-repeated claim that German silver acts germicidally in the mouth, but this he thought might prove a fertile field for scientific investigation. At all events the nobler metals being apparently cleaner and at the same time effective, it becomes the paramount duty of the specialist to finally determine by scientific experimentation what metals should best be used in orthodontic work. —Items of Interest.

Best Way in Inserting Inlays.—My best results in cementing porcelain fillings have been obtained by the following method.

First etch the surface to which the cement is to adhere, then cut with a knife edge disk irregular grooves around the bulbous portion of the filling, boil in a strong solution of sodium bicarbonate, and rinse in 95 per cent. alcohol. The soda is to prevent continued action of the acid, and the alcohol to free it from any fatty matter that may be acquired in the handling. That the action of hydrofluoric acid is continued for several hours unless this precaution be taken is easily proved by a thorough washing of the case after etching with brush and water and setting it aside for a day, or overnight. The result is a thick coating of a chalk-like substance covering the entire surface where the acid has been applied; this is easily removed with a small brush. Should the filling be inserted before the removal or elimination of this substance, whice of course spoils or prevents the adhesion of the cement, the result will be the loss of the filling within a few days.

The cement should be mixed to a creamy consistence, carefully spatulated to thoroughly incorporate the powder into the fluid and make it perfectly smooth; and with a fine-pointed instrument it is worked into all the undercuts of both cavity and filling; then the filling is placed in position and ligated with a heavy waxed ribbon floss, the surplus cement removed, a thick coating of sandarac varnish spread over the whole, and the patient is dismissed with instructions to remove the ligatures and varnish at the end of two hours.—Dr. F. T. Van Woert, Cosmos.

Pyorrhea Alveolaris.—As a corollary to this subject, I wish to introduce a technique and a set of prophylactic instruments in the treatment of pyorrhea alveolaris. The gum tissues are dried with a hot air syringe after placing cottonoid pads over the salivary ducts and sponging them with adrenalin chlorid, this causing the contraction of the arteries and reducing the tendency to hemorrhage and the liability to the absorption and consequent toxic effects of the anesthetic ointment, which is composed of:

℞. Cocoa butter.................℥ iij

White vaselin................℥ v

Cocain.........................gr. xiv

Menthol.......................gr. xxiv

Oil peppermint.............m. x

Chloretone....................gr. ix

Phenol.........................m. ij M.

Sig.—Apply on gum tissues after drying.

The above is applied with the finger to the gums after a second drying; these tissues are dried again with warm air, and by a process of osmosis the anesthetic and antiseptic are carried into the epithelium and mucous glands. These glands become paralyzed in their effort to supply additional secretion in response to the reflex call to allay the inflammation caused by the irritation of the serumal tartar and additional attacks of bacteria.

The pyorrheal pockets are filled with the same mixture, introduced by means of a collapsible tube fitted with a small platinum point and allowed to remain five minutes before beginning the scaling operation. The instruments are constructed in the form of a drag file, made to fit the curves of the surfaces to be worked upon. Their efficiency will be appreciated upon using them with a pull motion. They must be used as a fulcrum, and not by pressing them against the tooth, for being made of semi-hardened steel, they easily break.—Dr. F. T. Van Woert, Cosmos.

